# A Systematic Review of Neurofeedback for the Management of Motor Symptoms in Parkinson’s Disease

**DOI:** 10.3390/brainsci11101292

**Published:** 2021-09-29

**Authors:** Krithika Anil, Stephen D. Hall, Sara Demain, Jennifer A. Freeman, Giorgio Ganis, Jonathan Marsden

**Affiliations:** 1 Peninsula Allied Health Centre, School of Health Professions, University of Plymouth, Derriford Road, Plymouth PL6 8BH, UK; jenny.freeman@plymouth.ac.uk (J.A.F.); jonathan.marsden@plymouth.ac.uk (J.M.); 2 Brain Research and Imaging Centre, Faculty of Health, University of Plymouth, Research Way, Plymouth PL6 8BU, UK; stephen.d.hall@plymouth.ac.uk (S.D.H.); giorgio.ganis@plymouth.ac.uk (G.G.); 3 School of Psychology, University of Plymouth, Drake Circus, Plymouth PL4 8AA, UK; 4 School of Health Sciences, University of Southampton, Highfield, Southampton SO17 1BJ, UK; sara.demain@soton.ac.uk

**Keywords:** Parkinson’s disease, neurofeedback, movement, neural network activity, electroencephalography, neuroimaging

## Abstract

Background: Neurofeedback has been proposed as a treatment for Parkinson’s disease (PD) motor symptoms by changing the neural network activity directly linked with movement. However, the effectiveness of neurofeedback as a treatment for PD motor symptoms is unclear. Aim: To systematically review the literature to identify the effects of neurofeedback in people with idiopathic PD; as defined by measurement of brain activity; motor function; and performance. Design: A systematic review. Included Sources and Articles: PubMed; MEDLINE; Cinhal; PsychoInfo; Prospero; Cochrane; ClinicalTrials.gov; EMBASE; Web of Science; PEDro; OpenGrey; Conference Paper Index; Google Scholar; and eThos; searched using the Population-Intervention-Comparison-Outcome (PICO) framework. Primary studies with the following designs were included: randomized controlled trials (RCTs), non-RCTs; quasi-experimental; pre/post studies; and case studies. Results: This review included 11 studies out of 6197 studies that were identified from the literature search. Neuroimaging methods used were fMRI; scalp EEG; surface brain EEG; and deep brain EEG; where 10–15 Hz and the supplementary motor area were the most commonly targeted signatures for EEG and fMRI, respectively. Success rates for changing one’s brain activity ranged from 47% to 100%; however, both sample sizes and success criteria differed considerably between studies. While six studies included a clinical outcome; a lack of consistent assessments prevented a reliable conclusion on neurofeedback’s effectiveness. Narratively, fMRI neurofeedback has the greatest potential to improve PD motor symptoms. Two main limitations were found in the studies that contributed to the lack of a confident conclusion: (1) insufficient clinical information and perspectives (e.g., no reporting of adverse events), and (2) limitations in numerical data reporting (e.g., lack of explicit statistics) that prevented a meta-analysis. Conclusions: While fMRI neurofeedback was narratively the most effective treatment; the omission of clinical outcome measures in studies using other neurofeedback approaches limits comparison. Therefore, no single neurofeedback type can currently be identified as an optimal treatment for PD motor symptoms. This systematic review highlights the need to improve the inclusion of clinical information and more robust reporting of numerical data in future work. Neurofeedback appears to hold great potential as a treatment for PD motor symptoms. However, this field is still in its infancy and needs high quality RCTs to establish its effectiveness. Review Registration: PROSPERO (ID: CRD42020191097)

## 1. Introduction

Parkinson’s disease (PD) is a progressive neurological disorder that primarily disrupts normal motor functioning and affects 1–2 in every 1000 individuals in the general population [1]. Motor symptoms such as bradykinesia, rigidity, and tremor have a severe negative impact on quality of life and increase the likelihood of mood disorders such as depression [2,3]. PD motor symptoms are in part caused by degeneration of nigrostriatal neurons that reduces dopamine availability in the basal ganglia. Therefore, the main treatment for PD motor symptoms are pharmaceuticals, such as levodopa, that increase dopaminergic stimulation within the basal ganglia. However, the efficacy of these pharmaceuticals often declines over time and there is an increased occurrence of adverse side effects, such as dyskinesias, which may be severely disabling after prolonged use [4]. Other non-pharmaceutical treatments include high-frequency deep-brain stimulation (DBS) of specific structures of the basal ganglia, such as the subthalamic nuclei or the globus pallidus internus, through implanted electrodes. However, DBS is an invasive method and risks peri-operative complications such as intracranial hemorrhage, infections, and skin erosions [5,6]. Furthermore, DBS is not always effective and still incurs some unwanted side effects such as dysarthria, depression, apathy and executive dysfunction [7,8]. The development of non-pharmaceutical and non-invasive approaches that improve PD motor symptoms and minimize unwanted side effects would be therefore a valuable adjunct to symptom management.

Neurofeedback is a non-pharmaceutical treatment that uses a brain–computer-interface (BCI), allowing individuals to learn voluntary self-regulation of brain activity using an external, real-time representation of that brain activity [9]. Although neurofeedback can be invasive (e.g., recording from DBS electrodes [10]), non-invasive approaches, such as electroencephalography (EEG; [11]) or functional magnetic resonance imaging (fMRI; [12]), are viable alternatives.

The pathological neural activity associated with PD motor symptoms, such as bradykinesia, can be measured using several approaches. For example, non-invasive fMRI studies, have highlighted reductions in blood flow in the supplementary motor area with movement preparation [13] whilst recording of local field potentials from DBS electrodes in the STN have highlighted an increase in bursts of beta (15–30 Hz) oscillation during movement-preparation in PD [14]. Simultaneous recordings of beta oscillations highlight that cortical oscillatory activity phase leads and thus potentially drives that in the basal ganglia [15,16,17,18]. This suggests that neurofeedback of pathological activity using fMRI, EEG or local field potentials measured using DBS electrodes may hold potential for improving associated PD motor symptoms.

Previous neurofeedback research in PD has used a variety of different methodologies and protocols. There is extensive variation in aspects such as training regime, brain signature and feedback approach, which complicates evaluation of neurofeedback efficacy as an intervention for PD. In order to progress in this field, there is a need to establish the current evidence base for neurofeedback application in PD and explore the relative efficacy of different protocols.

### Review Question and Objectives

Here, we review the literature to address the following question: what are the effects of neurofeedback in people with idiopathic Parkinson’s disease (PD); as defined by measurement of brain activity, motor function, and performance?

The objectives of the review are to determine:

1. The effectiveness of neurofeedback as a treatment for reducing PD motor symptom severity.
2. The importance of specific protocol parameters for effective and reliable neurofeedback in terms of training regime, targeted brain activity, delivery of brain activity feedback signal, and changes in brain activity.
3. The association between specific neurofeedback protocols and clinical outcomes.

## 2. Methods

This systematic review was conducted in accordance with the Joanna Briggs Institute (JBI) methodology for systematic review effectiveness and according to the PRISMA statement. This protocol was registered on PROSPERO (ID: CRD42020191097).

### 2.1. Ethical Considerations

This study is a systematic literature review and did not involve human nor animal data collection. Therefore, ethical approval was not required.

### 2.2. Inclusion Criteria

The inclusion criteria were developed using the PICO (problem/population, intervention, comparison, and outcome) framework [19].

#### 2.2.1. Population

The current review considered studies that included participants with idiopathic PD (as defined by a stated clinical diagnosis) of any duration or severity, who were at least 18 years of age. Studies that included participants with any atypical parkinsonism (i.e., neurodegenerative parkinsonism other than PD) were excluded. This review also excluded studies involving participants who had any secondary causes of parkinsonism, such as drug-induced parkinsonism or lesions; however, such studies were included if it was possible to individually separate and remove participants with atypical parkinsonism from the analysis.

#### 2.2.2. Intervention

The review considered all studies that examined neurofeedback designed as a treatment for PD motor symptoms. This included studies that examined participants’ success at neurofeedback without any measurement of clinical outcomes. Relevant studies included neurofeedback training using any protocol (i.e., targeted brain activity), duration, frequency, or intensity. Neurofeedback evaluation considered any target brain activity such as EEG, deep brain recording, and other brain imaging (e.g., fMRI/positron emission tomography (PET)), and involved measurements from any brain region.

#### 2.2.3. Comparison

This review considered studies that compared neurofeedback with a comparator intervention or usual care. For studies with no comparator, neurofeedback results were presented narratively and were not included in any meta-analysis.

#### 2.2.4. Outcomes

This review considered studies that included the following outcomes:

1. Immediate and long-term sustained changes in brain activity following neurofeedback.
2. Immediate and long-term sustained changes in motor function or performance as measured by physiology (e.g., electromyography (EMG)) and/or other objective clinical outcome measures such as the Unified Parkinson’s Disease rating scale (UPDRS) or a questionnaire assessment of PD symptoms.

In addition to the above outcomes, this review evaluated the following information for the narrative analysis:

1. Neurofeedback protocol (i.e., targeted brain activity, presentation of brain activity to participants, criteria for “successful” neurofeedback).
2. Neurofeedback training details (i.e., who provides the neurofeedback training, guidance provided to participants, training regime).
3. The relationship between the above neurofeedback details and neurofeedback outcomes (i.e., success at neurofeedback and clinical outcomes).

#### 2.2.5. Types of Studies

This review considered experimental study designs including randomized controlled trials (RCTs), non-RCTs, quasi-experimental, pre/post studies, and case studies. Only studies in English were included. Animal studies, observational studies, and narrative studies were excluded.

### 2.3. Search Strategy

The search strategy aimed to find both published and unpublished studies. A three-step search strategy, developed in discussion with a data-synthesis specialist, was utilised. An initial limited search of PubMed was undertaken to estimate the volume of relevant literature and to identify key words to assist in developing search terms. A second search using the developed search terms was undertaken and adapted across each included information source (see Supplementary Material “SM1—Search Strategy”); this included searches for published and grey literature. The third strategy involved searching for additional studies within the reference list of all studies that met the inclusion criteria. In cases of ongoing studies, authors were contacted for further information to determine eligibility for inclusion in this review. No limiters were used.

### 2.4. Information Sources

The databases searched were: PubMed, MEDLINE, Cinhal, PsychoInfo, Prospero, Cochrane, ClinicalTrials.gov, EMBASE, Web of Science, PEDro, OpenGrey, Conference Paper Index, Google Scholar, and eThos.

### 2.5. Study Selection

**Figure 1:**
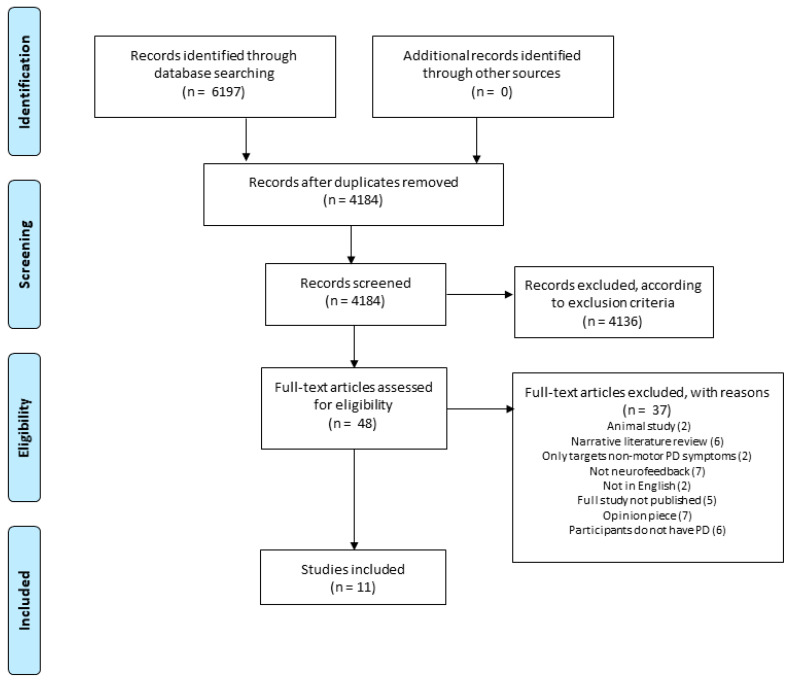
PRISMA flowchart for the literature search process.

All identified references were imported into citation software (EndNote, Clarivate Analytics [20]). Duplicates were removed before uploading to the online collaborative systematic review organization tool ‘Raayan’ [21]. Titles and abstracts were screened by two reviewers (KA and JM) independently against the review inclusion criteria. The full-text of potentially eligible studies were retrieved and assessed in detail against the inclusion criteria by the reviewers (See Figure 1 for the PRISMA flowchart. Reviewer discrepancies were resolved through discussion with a third reviewer when necessary.

### 2.6. Assessment of Methodological Quality

Eligible studies were critically appraised for methodological quality by two independent reviewers using the standardized JBI critical appraisal instruments. Any disagreements were resolved through discussions, and through a third reviewer if necessary. All studies were included regardless of methodological quality. Originally, poor quality studies were planned to be excluded. However, majority of the studies were not of high quality and the decision was made to include all studies that met the eligibility criteria to report on the current status of this field. The results of critical appraisal are reported in Table 1 in the Section 3.

### 2.7. Data Extraction

Data extraction of included studies was conducted by the same reviewers (KA and JM). Narrative data extraction was conducted using an Excel spreadsheet. The following data were extracted for narrative synthesis: study design, study objectives, attrition details, demographics, PD symptoms, medication details, target brain activity, feedback signal delivery, and training (e.g., who provided the neurofeedback training, guidance provided, training regime), assessment of neurofeedback performance, success rates, clinical measures and outcomes (if any), and follow-up details (if any). Data extraction for a meta-analysis was planned (see protocol on PROSPERO, ID: CRD42020191097); however, the lack of numerical data in the identified articles prevented the meta-analysis.

### 2.8. Data Synthesis

The narrative synthesis of findings from included studies was structured according to the review objectives. As statistical pooling was not possible due to the poor reporting of numerical data (see the Section 3 and Section 4 for details), the findings were presented narratively aided by appropriate tables and figures. Publication bias investigation was not possible as the meta-analysis could not be conducted.

### 2.9. Assessing Certainty in Findings

A Summary of Findings (SoF) table was developed and includes the following outcomes: changes in brain activity, neurofeedback success rates, changes in movement and motor function, and adverse events. Further outcomes could not be included as planned due to inadequate reporting of numerical data. These were: absolute risks for the treatment and control, estimates of relative risk and evaluation of bias, directness, heterogeneity, and precision.

## 3. Results

Eleven studies were included (Figure 1). Table 1 shows the methodological assessments conducted using the relevant JBI critical appraisal instruments, dependent on the study design. Please see Supplementary Material “SM2—JBI Methodological Assessment Items”. Overall, most studies were of low quality with only one study (Fukuma et al. [22]) of high quality, scoring 89%. The main reason for low quality was the lack of appropriate follow-ups. However, several studies did not include various methodological details (e.g., demographic or study condition information), scoring “unclear” on these items. This low quality is reflected in these studies overall lack of numerical data.

Table 2 shows the study characteristics: five examined changes in both brain activity and movement; the remaining six only examined brain activity changes. Three studies included a control condition, of which only one study blinded participants using a sham trial [23]. Participants were partially blinded in one study that used a crossover design only for participants in the control condition, who were subsequently un-blinded when they crossed over to the neurofeedback condition [24]. Assessors were blinded in only one of these three studies [25]. Table 1 shows that only 2 studies specified the PD symptom they were targeting: Buyukturkoglu et al. [26] targeted hand-motor symptoms and akinesia, while Erikson-Davis et al. [24] targeted levodopa-induced dyskinesia. The remaining 9 studies reported general PD symptoms or did not mention their target.

The change in clinical outcomes with NF training are summarised in Table 3, the lack of available data and variability of the contributing studies precluded a meta-analysis resulting in a narrative report of the findings. Table 3 shows that PD severity was defined using the Hoehn and Yahr scale (3 studies [23,26,27]), the United Parkinson’s disease Rating scale (UPDRS) reported in full (2 studies [25,29]), or using the motor subsection of the UPDRS (4 studies [22,24,30,32]). Two studies did not measure PD severity [28,31]. Clinical outcomes included reaction times and finger tapping tests [23,26], EMG amplitude [22], UPDRS [23,25,29], diaries [24], and questionnaires (Modified Abnormal Involuntary Movement Scale [24] and PDQ-39 [25]). The three studies [23,25,29] that used the UPDRS as a clinical outcome reported a reduction in the score in the intervention group (indicating an improvement in clinical outcome) ranging from −0.3 [29] to −5.2 [23]. Mean changes in the primary outcome or measures of outcome variability (e.g., standard deviation) were not reported in 3 studies [22,23,24], while 5 studies did not measure any clinical outcome [27,28,30,31,32]. Adverse events were not reported in any study.

Table 4 displays details of the neurofeedback training protocol and regimes from the included articles. Four types of brain imaging were used for neurofeedback: scalp EEG (Erickson-Davis et al. [24]; Fumuro et al. [27]; Kasahara et al. [30]; Thompson & Thompson [31]), deep brain stimulation EEG using local field potentials (Fukuma et al. [22]; Khanna & Carmena [32]), electrocorticography (ECoG; (He et al. [28])), and fMRI (Buyukturkoglu et al. [26]; Subramanian et al. [23]; Subramanian et al. [25]; Tinaz et al. [29]). The most commonly used was scalp EEG and fMRI. The control direction for targeted brain activity varied amongst the studies; however, 10–15 Hz was the most commonly targeted EEG activity and SMA activation with movement preparation was the most commonly targeted fMRI activity. Success rates (i.e., the rate of participants able to change their brain activity in the desired direction) ranged from 47% to 100%; however, both sample sizes and success criteria differed between studies. Study-defined success criteria were based on brain activity, comparing brain activity power during neurofeedback to baseline or comparing baseline power between pre and post neurofeedback training. One study based its criteria on EEG amplitude threshold (Fumuro et al. [27]), while another identified success by predicting if seemingly successful performances were by chance by comparing it to simulated performances (Khanna & Carmena [32]). Only one study provided audio feedback of brain activity (i.e., a tone sounded when performance was successful) (Erickson-Davis et al. [24]), while the remaining 10 provided visual feedback. The visual feedback was divided into three categories: changes in bar height (Buyukturkoglu et al. [26]; Subramanian et al. [23]; Subramanian et al. [25]; Tinaz et al. [29]), object moves (e.g., up and down) (Fumuro et al. [27]; He et al. [28]; Kasahara et al. [30]; Khanna & Carmena [32]), and object changes in size (i.e., bigger or smaller) (Fukuma et al., 2018 [22]). The bar changing in height was sometimes a solid-colored rectangle, but was commonly a “thermometer” that had height indicators (see figures in Subramanian et al., (Subramanian et al. [23]; Subramanian et al. [25]) for examples). The “object” in the remaining two categories was of various designs, such as a ball or a video game character. No justification was provided for choosing a delivery design in any study. Specific instructions for controlling brain activity were provided in 6 of the 11 studies (Buyukturkoglu et al. [26]; He et al. [28]; Kasahara et al. [30]; Subramanian et al. [23]; Subramanian et al. [25]; Tinaz et al. [29]), all of which involved motor imagery. No specific instructions were provided in 3 of the studies (Erickson-Davis et al. [24]; Fukuma et al. [22]; Fumuro et al. [27]), while the remaining 2 studies did not describe the instructions (Khanna & Carmena [32]; Thompson & Thompson [31]). Neurofeedback runs ranged from 10 s to 12 min, while neurofeedback sessions ranged from 10 min to 50 min. The number of sessions ranged from 1 to 42 sessions, and time between sessions ranged from 1 day to 6 months.

Table 5 provides a summary of the targeted brain activity, success rates, and whether neurofeedback success was accompanied by a change in clinical outcome measures. No studies to date have assessed the long-term follow up of neurofeedback effects. Using fMRI neurofeedback of SMA activity and right insula-dorsomedial frontal cortex functional connectivity were the only approaches that achieved both success in neurofeedback and a change in clinical outcome, as measured by the UPDRS motor scale in all studies (Subramanian et al. [23]; Subramanian et al. [25]; Tinaz et al. [29]), and additionally finger tapping (Subramanian et al. [23]) or the PDQ-39 (Subramanian et al. [25]).

## 4. Discussion

The 11 studies included in this systematic review highlighted that the development of a neurofeedback intervention for PD motor symptoms is still in its early stages. Neurofeedback studies using fMRI as a measurement approach appear to report the highest success rates compared to other measurement approaches; however, two of these studies were generated by the same research team (i.e., Subramanian et al. [23,25]) and would therefore benefit from external validation. Six of the 11 studies [22,23,24,25,26,29] included a clinical outcome (4 of which used fMRI neurofeedback [23,25,26,29]). The limited number of comparable methodological approaches limits the confidence in any conclusion about the most effective type of neurofeedback for modulation of PD motor symptoms. The inability to draw confident conclusions on the effectiveness of neurofeedback as a modulator of PD motor symptoms, concurs with Esmail and Linden’s [33] systematic review on the therapeutic value of neurofeedback for PD and suggests that this field has seen little advancement in the years since 2014. We identified two principal reasons for this limited progress: (1) insufficient clinical information and perspectives, and (2) limitations in numerical data reporting.

### 4.1. Insufficient Clinical Information and Perspectives

Six studies assessed clinical outcomes [22,23,24,25,26,29] whilst the remaining studies assessed the feasibility of achieving neurofeedback according to specific success criteria. The primary aim of 5 out of 6 studies investigating clinical outcomes was an improvement in the hypokinetic motor symptoms related to PD. In contrast, Erikson-Davis et al. [24] investigated the effects of neurofeedback on reducing Levodopa-induced dyskinesias. The proposed pathophysiological mechanisms of hypo- and hyper-kinetic deficits in PD vary. Animal and human studies suggest increased bursts of beta oscillations cause hypokinetic deficits [14,34,35,36]. In contrast, dyskinesias resulting from either long term dopamine replacement therapy or DBS are associated with an increase in 4–10 Hz oscillations within the basal ganglia-cortical region [37,38]. Reflecting this, studies using EEG and DBS to target hypokinetic symptoms aimed to reduce beta-band activity through neurofeedback whilst the study targeting dyskinesia aimed to primarily reduce 4–8 Hz activity through neurofeedback [24]. Thus, when using EEG or DBS based neurofeedback, the underlying targeted symptoms should define the spectral band of interest. Although the role of oscillatory activity in the control of normal movements remains under investigation [39,40]. The fact that oscillatory activity is associated with normal movement suggests that a gross reduction in oscillatory power across a certain spectral band in PD may not be the optimal approach. Recent work has highlighted the importance of bursts of beta oscillations in the development of bradykinesia [15,41,42] and future work should target the incidence of these abnormal busts rather than gross changes in oscillatory power. Studies in healthy participants show that this is possible [43].

The optimal target and mechanism for recording brain activity to provide neurofeedback in PD cannot be determined from the review. The strongest evidence provided is for neurofeedback of SMA activity using fMRI. This is supported by a review [13] highlighting the significant reductions in blood flow in the supplementary motor area with movement preparation in PD. However, such changes are not seen in all studies. Although a promising area of research, the use of fMRI may be difficult to implement as an intervention given issues such as expense, availability and technical demands. Whilst fMRI has a good spatial resolution, its temporal resolution is low, resulting in a smearing of information in the time-frequency domain. Therefore, while anatomical resolution is high, it has limited ability to resolve specific phases of the movement process or individual neural signatures involved. In contrast, EEG has poor spatial and good temporal resolution. Therefore, it is capable of resolving discrete phases of movement and neural signatures (e.g., frequency bands), however, spatial smearing means that the target for EEG in the current studies could reflect an amalgamation of activity across primary and secondary motor areas (e.g., SMA). EEG as a neurofeedback device has potential advantages in terms of costs and ease of use with the possibility of home-based training. Simultaneous recordings of beta oscillations at rest highlight that cortical oscillatory activity phase leads and thus potentially drives that in the basal ganglia [15,16,17,18,19]. However, beta power is higher in the STN compared to the cortex, reflecting the fact that beta activity may be amplified within the basal ganglia [44] alongside the low pass filtering effects of the motor cortex on re-entrant beta oscillations [45]. This suggests that targeting activity within the basal ganglia nuclei may be preferable. This, however, requires access to surgically implanted DBS electrodes. Understanding the effects of both increasing and decreasing motor cortical spectral power and oscillatory bursts within motor cortical areas on movement performance in participants with and without PD could inform the role of cortical oscillations in the control of movement, and thus the potential role of EEG as a target for neurofeedback.

### 4.2. Limitations in Numerical Data Reporting

Examples of limitations in the reporting of numerical data includes a lack of reporting of basic descriptive statistics, such as measures of central tendency and variance required to demonstrate the magnitude of change between time points, study conditions, and neurofeedback parameters. Some studies did not report measures of statistical significance, such as *p*-values, and others failed to identify the statistical tests applied. This reflects the exploratory nature of some papers in this review. The absence of this information prevents the drawing of meaningful conclusions for individual studies and prevented meta-analysis intended to enrich this review. Additionally, lack of information about statistical power calculations (as well as small sample sizes) further restricted the drawing of meaningful conclusions. Limited reporting of numerical data confines the speed and reliability of progress in the development of neurofeedback for PD motor symptoms. Consequently, in this paper we strongly suggest adherence to CONSORT reporting standards for quantitative data. In particular, we suggest closely following the sections detailed in Table 6 below.

Ros et al. [46] have produced a checklist for reporting experimental neurofeedback studies, which helps authors to clearly outline their study design and analysis. We therefore recommend adhering to both the CONSORT guidance (especially focusing on the guidance in Table 5) and the checklist by Ros et al. [46] to ensure high quality reporting of neurofeedback for PD.

### 4.3. Study Limitations

There were several limitations to the review. No meta-analysis was undertaken due to limitations in the data presented and heterogeneity in the papers reviewed. Further, there was an English language bias in the papers reviewed. Ongoing studies of neurofeedback in Parkinson’s disease have been identified from clinicaltrials.org (e.g., NCT03837548). These will be published after this paper has been published, and may change this review’s outcome

## 5. Conclusions

This systematic review highlights the need to improve the reporting of numerical data and neurofeedback parameters in future work. Although based on a few studies there is a suggestion that fMRI-based neurofeedback may be effective and be associated with an improvement in hypokinetic symptoms. It is unclear whether EEG based neurofeedback is effective in PD. The targeted spectral band would vary depending on the symptoms of interest (dyskinesia vs. hypokinesia). Further work should investigate the role of cortical oscillations in driving abnormal oscillatory activity in the Basal Ganglia and whether EEG could be a potential target for neurofeedback.

## Figures and Tables

**Table 1:** Methodological assessment of included studies using critical appraisal instruments from the Joanna Briggs Institute.

**Quasi Experimental Studies**	**Q1**	**Q2**	**Q3**	**Q4**	**Q5**	**Q6**	**Q7**	**Q8**	**Q9**	* Total				
Buyukturkoglu et al. [26]	Y	N	N	N	Y	N	N	U	Y	33%				
Fukama et al. [22]	Y	Y	Y	Y	Y	N	Y	Y	Y	89%				
Fumuro et al. [27]	Y	U	Y	N	Y	N	Y	Y	Y	67%				
He et al. [28]	Y	U	U	N	Y	Y	Y	U	Y	56%				
Subramanian et al. [23]	Y	U	U	Y	Y	N	Y	Y	Y	67%				
Tinaz et al. [29]	Y	Y	U	Y	U	N	N	Y	Y	56%				
**RCTs**	**Q1**	**Q2**	**Q3**	**Q4**	**Q5**	**Q6**	**Q7**	**Q8**	**Q9**	**Q10**	**Q11**	**Q12**	**Q13**	* Total
Erikson-Davis et al. [24]	U	U	N	N	N	U	N	N/A	Y	Y	Y	N	N	23%
Subramanian et al. [25]	Y	N	U	N	N	Y	N	N	Y	N	Y	Y	N	38%
**Case reports**	**Q1**	**Q2**	**Q3**	**Q4**	**Q5**	**Q6**	**Q7**	**Q8**	* Total					
Kasahara et al. [30]	Y	N	Y	Y	Y	N	N	N	50%					
Thompson & Thompson [31]	N	N	Y	N	Y	Y	U	N	38%					
**Cross-sectional studies**	**Q1**	**Q2**	**Q3**	**Q4**	**Q5**	**Q6**	**Q7**	**Q8**	* Total					
Khanna & Carmena [32]	N	N	Y	N	N	N	Y	Y	38%					

Q = Question; Y = Yes; N = No; U = Unclear; RCT = Randomised controlled trial; N/A = Not applicable; * Total refers to the percentage of “yes” answers for each study, where a higher percentage indicates a higher quality study.

**Table 2:** Study characteristics.

Author	Aim	Targeted PD Symptom	Country	Design	Intervention Condition	Control Condition	Total N
Buyukturkoglu et al., (2013) [26]	Examining the effectiveness of real-time fMRI neurofeedback (reinforcement of SMA BOLD signal) on hand motor performance	Hand-motor performance/akinesia	USA	Case study	fMRI neurofeedback, finger tapping	No control condition	1
Erikson-Davis et al., (2012) [24]	Testing if scalp EEG neurofeedback (reinforcement of 12–15 Hz, suppression 4–10 Hz and 11–30 Hz) would lead to a decrease in PD motor-symptoms	Levodopa-induced dyskinesia	USA	RCT	Scalp EEG neurofeedback	Sham trial, followed by scalp EEG neurofeedback	9
Fukama et al., (2018) [22]	Examining if DBS EEG neurofeedback (reinforcement and suppression of 13–30 Hz from STN) induces plastic changes in the STN activity of individuals with PD	General motor symptoms	Japan	Quasi-experimental	DBS neurofeedback	No control condition	8
Fumuro et al., (2013) [27]	Examining whether PD patients could increase BP amplitude with scalp EEG neurofeedback	No target symptom reported	Japan	Quasi-experimental	Scalp EEG neurofeedback	No control condition	21
He et al., (2019) [28]	Investigating whether DBS EEG neurofeedback (supress beta rhythms in STN) is possible for people with PD	General motor symptoms	UK	Observational	DBS EEG neurofeedback	No control condition	3
Kasahara et al., (2018) [30]	Examining scalp EEG neurofeedback (reinforcement and suppression 9.5–12.5 Hz of SMR) in a patient with PD	No target symptom reported	Japan	Case study	Scalp EEG neurofeedback, motor imagery practice	No control condition	1
Khanna & Carmena (2017) [32]	To show that PD patients can control beta activity using DBS EEG neurofeedback	General motor symptoms	USA	Observational	DBS neurofeedback	No control condition	3
Subramanian et al., (2011) [23]	Assessing whether PD patients are able to alter local brain activity to improve motor function	General motor symptoms	UK	Quasi-experimental	fMRI neurofeedback, hand movement task, home practice of motor imagery	Sham trial, hand movement task, home practice of motor imagery	10
Subramanian et al., (2016) [25]	Determining the effect of neurofeedback and motor training alone on motor and non-motor functions in PD	General motor and non-motor symptoms	UK	RCT	fMRI neurofeedback, hand motor task, Wii fit motor training, home practice of motor imagery	Wii fit motor training	30
Thompson & Thompson (2002) [31]	To present a theoretical framework for a biofeedback treatment for movement disorders using a case study involving dystonia with PD	General motor symptoms	Canada	Case study	Scalp EEG, RSA training	No control condition	1
Tinaz et al., (2018) [29]	Testing the ability of those with PD to learn to use fMRI neurofeedback (reinforcement of the right insula-dorsomedial frontal cortex functional connectivity)	General motor symptoms	USA	Quasi-experimental	fMRI neurofeedback, motor imagery practice, heartbeat counting task, home practice of motor imagery	No control condition	8

PD = Parkinson’s Disease; RCT = Randomised Controlled Trial; RSA = respiratory sinus arrhythmia; SMA = Supplementary Motor Area; BP = Bereitschaft potential; STN = Subthalamic Nucleus; SMR =Sensorimotor rhythm.

**Table 3:** Details of clinical outcome measures.

Author(s) (Date)	PD Severity Measure	Mean (SD/Range)	Outcome Measure	Change within the Intervention Condition	Change within the Control Condition	Relative Change ^a^ Between Conditions
Buyukturkoglu et al., (2013) [26]	Hoehn and Yahr Scale	2.5 (SD not reported)	Button pressing reaction time in seconds	23 (±83)	No control condition	N/A
Erikson-Davis et al., (2012) [24]	UPDRS-III	20 (4–42)	Parkinson’s Disease Home Diary	0 (SD not reported)	2 (SD not reported)	Insufficient data for calculation
			Modified Abnormal Involuntary Movement Scale	−2.5 (SD not reported)	−2 (SD not reported)	Insufficient data for calculation
Fukama et al., (2018) [22]	UPDRS-III	31.13 (±20.49)	Pre-post EMG resting baselines	Not reported **	No control condition	N/A
Fumuro et al., (2013) [27]	Hoehn and Yahr Scale	Not reported	None	N/A	N/A	N/A
He et al., (2019) [28]	None	N/A	None	N/A	N/A	N/A
Kasahara et al., (2018) [30]	UPDRS-III	13 (SD not reported)	None	N/A	N/A	N/A
Khanna & Carmena (2017) [32]	UPDRS-III	Not reported	None	N/A	N/A	N/A
Subramanian et al., (2011) [23]	Hoehn and Yahr Scale	1.3 (±0.64)	UPDRS—Motor Scale	−5.2 (SD not reported) **	−1.6 (SD not reported)	4.4 (SD not reported) ^b^
			Finger tapping test	55.6 (SD not reported) **	1.2 (SD not reported)	−88 (SD not reported) ^b^
Subramanian et al., (2016) [25]	UPDRS	25 (±11)	UPDRS—Motor Scale	−4.5 (±3.3) ***	−1.8 (±8.3)	Sufficient data not available for calculation
			PDQ-39	−2.4 (±4.8) *	−3.6 (±6.5)	Sufficient data not available for calculation
Thompson & Thompson (2002) [31]	None	N/A	None	N/A	N/A	N/A
Tinaz et al., (2018) [29]	UPDRS	44.8 (±5.4)	UPDRS—Motor Scale	−0.3 (±2.1)	No control condition	N/A

^a^ Change between final measurements of the control condition and the intervention condition (i.e., condition—intervention = relative change); ^b^ Analysis conducted by review authors, not authors of original study; thus, there is no *p* value for this result; PD = Parkinson’s disease; UPDRS = Unified Parkinson Disease Rating Scale; PDQ = Parkinson’s Disease Questionnaire; ON/OFF = Refers to whether patients were on or off their medication when completing the outcome measure; N/A = Not applicable; * *p* value < 0.1; ** *p* value < 0.05; *** *p* value < 0.01.

**Table 4:** Details of neurofeedback training, regime, and success.

Paper	NF Type	NF Targeted Activity	NF Run Length	NF Session Length	No. Sessions	Time between Sessions	Delivery Method	Instructions Given on How to Complete the Task	Success Criteria?	Success Rates
Buyukturkoglu et al., (2013) [26]	fMRI	SMA Reinforcement	22.5 s	3–4 (Varied between participants)	1–2 (Varied between participants)	5 days	Thermometer (A vertical bar with height targets)	Motor imagery	Not reported	100%
Erikson-Davis et al., (2012) [24]	Scalp EEG	C3 & C4 Reinforce 8–15 Hz Inhibit 4–8 Hz Inhibit 23–34 Hz	Not reported	30 m	24	1–6 days	Audio feedback	No specific instructions	Not reported	Not reported
Fukama et al., (2018) [22]	DBS EEG	STN Reinforce or inhibit 13–30 Hz	10 m	10 m	1	N/A	Circle whose size changed with 13–30 Hz power changes	No specific instructions	Change in pre post EEG levels as determined by *t* test	75%
Fumuro et al., (2013) [27]	Scalp EEG	Cz Bereitschaftspotential	10 s	8.7 m	2–4	1–6 days	A sunfish moved up or down depending on potential shift	No specific instructions	Amplitude must have exceeded a defined target level (based on baseline) and remained at that level for at least 2 s in the last 4 s of each trial	40% and 45% for PD and control groups, respectively
He et al., (2019) [28]	ECoG	Left or Right STN Inhibit 13–30 Hz	5–8 s	30 m	1	N/A	A basketball moved vertically, where the basketball went higher with reduced beta power	Motor imagery of hand	Comparing ball position between neurofeedback training and no neurofeedback training sessions	66%
Kasahara et al., (2018) [30]	Scalp EEG	C3 or C4 Reinforce and inhibit 9.5–12.5 Hz	4 s	24 min	2 (ON and OFF)	2 days	A falling cursor that moved left or right to hit a target depending on targeted ERD	Motor imagery of the left or right hand	Ability to hit target	On medication 65% Off medication 58%
Khanna & Carmena (2017) [32]	DBS EEG	STN Reinforce and inhibit 13–30 Hz	5–15 m	25–150 m	1	NA	A video game character (Mario) moved according to 13–30 Hz power	Not reported	Comparing actual performance over time to simulated performance over time to determine if actual performance exceeded distribution of chance simulated performance	100%
Subramanian et al., (2011) [23]	fMRI	SMA reinforcement	20 s	13 m	2	2–6 months	Thermometer (A vertical bar with height targets)	Motor imagery suggested	Statistically significant increase in SMA activity compared to baseline	100%
Subramanian et al., (2016) [25]	fMRI	SMA reinforcement	20 s	12 min	3	1–4 weeks	Thermometer (A vertical bar with height targets)	Motor imagery suggested	Positive “t” or “beta” value for the increase in SMA activity compared to baseline	Success rate for individuals not reported
Thompson & Thompson (2002) [31]	Scalp EEG	FCz-CPz or Cz Reinforce 13–15 Hz Inhibit 9–10 Hz Inhibit 25–32 Hz	Not reported	50 m	42	1 week	Not reported	Not reported	Not reported	Not reported
Tinaz et al., (2018) [29]	fMRI	Right insula-dorsomedial frontal cortex functional connectivity reinforcement	8 s	6.7–8 m	2	1–2 weeks	A bar plot, where a blue bar indicated negative brain activity and a red bar indicated positive brain activity	Motor Imagery	Significant increase in brain connectivity of pre-post baseline scans	Success rate for individuals not reported

ON/OFF = Refers to whether patients were on or off their medication when completing the neurofeedback task; NF = Neurofeedback; fMRI = Functional magnetic resonance imaging; EEG = Electroencephalography; DBS = Deep brain stimulation; STN = Subthamalic nucleus; SMA = Supplementary motor area; N/A = Not applicable.

**Table 5:** Treatment summary table.

Author(s) (Date)	NF Type	Targeted Activity	Activity Direction	Clinical Outcome Improved?	NF Achieved?	Indicative * Support for NF Treatment?	Follow-Up?
Buyukturkoglu et al., (2013) [26]	fMRI	SMA	Reinforcement	No	Yes	No	No
Erikson-Davis et al., (2012) [24]	Scalp EEG	C3 & C4 8–15 Hz 4–8 Hz 23–34 Hz	Both suppression and reinforcement	No	Not reported	No	No
Fukama et al., (2018) [22]	DBS EEG	STN 13–30 Hz	Both suppression and reinforcement	No	Partially (75% successful)	No	No
Subramanian et al., (2011) [23]	fMRI	SMA activity	Reinforcement	Yes	Yes	Yes	No
Subramanian et al., (2016) [25]	fMRI	SMA activity	Reinforcement	Yes	Yes	Yes	No
Tinaz et al., (2018) [29]	fMRI	Right insula-dorsomedial frontal cortex functional connectivity	Reinforcement	Yes	Yes	Yes	No

NF = Neurofeedback; fMRI = Functional magnetic resonance imaging; EEG = Electroencephalography; DBS = Deep brain stimulation; STN = Subthamalic nucleus; SMA = Supplementary motor area; * This support was based on whether there was a “yes” in both the “clinical outcome improve?” and the “NF achieved?” columns. As a meta-analysis could not be conducted, this support is only indicative and not conclusive.

**Table 6:** Specific sections that should be followed by studies developing neurofeedback for PD motor symptoms.

No.	Section Name	Description	Reason for Suggestion
7a	Sample Size	Sample size determination and/or calculations	No studies included a sample size calculation nor a justification for their recruitment sample size.
12	Statistical Methods and Additional Analysis	Statistical methods used for all outcome measures and any additional analysis	Many studies did not clearly report (or did not report at all) the statistical tests used nor the justification for these tests. Furthermore, many studies excluded vital information regarding means, standard deviations, error data, or *p*-values.
13–18	These sections all refer to results reporting	Beyond reporting outcomes measures, these sections also refer to important information such as participant flow, recruitment, baseline data, and sample size that was analysed	The results section of many studies excluded vital information needed for a meta-analysis and drawing a meaningful conclusion. Specifically, information regarding neurofeedback success rates are needed (e.g., individual success rates and success thresholds).
19	Harms	Any adverse events or unintended effects	Any treatment development must monitor side effects. No study reported this monitoring as part of their study process.

## Data Availability

No data available as this was a systematic review.

## Supplementary Materials

The following are available online at https://www.mdpi.com/article/10.3390/brainsci11101292/s1.
