# Supplementary material for: A Systematic Review of Neurofeedback for the Management of Motor Symptoms in Parkinson’s Disease
Source: Brain Sci. 2021 Sep 29;11(10):1292. doi: 10.3390/brainsci11101292 (PMC8534214; doi:10.3390/brainsci11101292)
Supplement: Supplementary file 1 [file brainsci-11-01292-s001.zip › SM1 Search Strategy.pdf]

Supplementary Material: Search Strategy and Results

Search Strategy

The initial search produced limited hits, suggesting that the search strategy should be broadened to increase sensitivity and reduce the risk of accidental exclusion of relevant studies. The below search strategy used a logic grid and is based on the PI (population and intervention) of PICO. The first and second column were combined for all searches, where possible. The third column ("target") represents specific details of the intervention. As advised by an evidence synthesis specialist, the third column was only used to narrow searches that produced an overwhelming quantity of results.

**Population**	**Intervention**	**Target**
Parkinson*	Neurofeedback	fMRI
	Neuro feedback	EEG
	Feedback	Deep brain
	Biofeedback	Subthalamic nucleus
	BCI	Globus pallidus
	Brain computer interface	Encephalography
		Brain imaging
		PET

Search Strategy Results

Database: PubMed

Date: 22/07/2020

ID	Search Term(s)	Hits
1	Parkinson*	134,810
2	(neurofeedback) OR (neuro feedback) OR (biofeedback) OR (feedback) OR (BCI) OR (brain computer interface)	175,352
3	ID 1 AND ID 2	1,246

Database: CINHAL

Date: 22/07/2020

ID	Search Term(s)	Hits
1	Parkinson*	30,715
2	(neurofeedback) OR (neuro feedback) OR (biofeedback) OR (feedback) OR (BCI) OR (brain computer interface)	45,928
3	ID 1 AND ID 2	250

Database: PsychInfo

Date: 23/07/2020

ID	Search Term(s)	Hits
1	Parkinson*	37,944
2	(neurofeedback) OR (neuro feedback) OR (biofeedback) OR (feedback) OR (BCI) OR (brain computer interface)	82,445
3	ID 1 AND ID 2	565

Database: Prospero (Completed only)

Date: 04/08/2020

ID	Search Term(s)	Hits
1	Parkinson* AND neurofeedback	1
2	Parkinson* AND neuro feedback	0
3	Parkinson* AND biofeedback	0
4	Parkinson* AND feedback	7
5	Parkinson* AND BCI	1
6	Parkinson* AND brain computer interface	1

Database: Cochrane (Reviews only)

Date: 04/08/2020

ID	Search Term(s)	Hits
1	Parkinson* AND (neurofeedback OR neuro feedback OR biofeedback OR feedback OR BCI OR brain computer interface)	0

Database: ClinicalTrials.gov (Completed only)

Date: 04/08/2020

ID	Search Term(s)	Hits
1	Parkinson* AND (neurofeedback OR neuro feedback OR biofeedback OR feedback OR BCI OR brain computer interface)	7

Database: EMBASE

Date: 04/08/2020

ID	Search Term(s)	Hits
1	Parkinson*	208,631
2	neurofeedback OR neuro feedback OR biofeedback OR feedback OR BCI OR brain computer interface	228,151
3	ID 1 AND ID 2	2217

Database: Web of Science

Date: 04/08/2020

ID	Search Term(s)	Hits
1	TS=Parkinson*	175,953
2	TS=(neurofeedback OR neuro feedback OR biofeedback OR feedback OR BCI OR brain computer interface)	467,398
3	ID 1 AND ID 2	1,891

Database: PEDro

Date: 19/08/2020

ID	Search Term(s)	Hits
1	Parkinson* AND neurofeedback	3
2	Parkinson* AND neuro feedback	0
3	Parkinson* AND biofeedback	6
4	Parkinson* AND BCI	0
5	Parkinson* AND brain computer interface	0

Database: OpenGrey

Date: 19/08/2020

ID	Search Term(s)	Hits
1	(Parkinson*) AND (neurofeedback OR neuro feedback OR biofeedback OR feedback OR BCI OR brain computer interface)	3

Database: eThos

Date: 19/08/2020

ID	Search Term(s)	Hits
1	Parkinson* AND neurofeedback	3
2	Parkinson* AND neuro feedback	0
3	Parkinson* AND biofeedback	2
4	Parkinson* AND BCI	2
5	Parkinson* AND brain computer interface	1

Database: Google Scholar

Date: 20/08/2020

ID	Search Term(s)	Hits
1	Parkinson* neurofeedback|neuro feedback|biofeedback|feedback|BCI|brain computer interface	33,600

Only the first 200 hits were scanned
