# Supplementary material for: A Systematic Review of Neurofeedback for the Management of Motor Symptoms in Parkinson’s Disease
Source: Brain Sci. 2021 Sep 29;11(10):1292. doi: 10.3390/brainsci11101292 (PMC8534214; doi:10.3390/brainsci11101292)
Supplement: Supplementary file 1 [file brainsci-11-01292-s001.zip › SM2 JBI Methodological Assessment Items.pdf]

JBI Methodological Assessment Items

Items for Quasi-Experimental Studies

No.	Question
Q1	Is it clear in the study what is the 'cause' and what is the 'effect' (i.e., there is no confusion about which variable comes first)?
Q2	Were the participants included in any comparisons similar?
Q3	Were the participants included in any comparisons receiving similar treatment/care, other than the exposure or intervention of interest?
Q4	Was there a control group?
Q5	Were there multiple measurements of the outcome both pre and post the intervention/exposure?
Q6	Was follow up complete and if not, were differences between groups in terms of their follow up adequately described and analyzed?
Q7	Were the outcomes of participants included in any comparisons measured in the same way?
Q8	Were outcomes measured in a reliable way?
Q9	Was appropriate statistical analysis used?

Items for Randomised Controlled Trials

No.	Question
Q1	Was true randomization used for assignment of participants to treatment groups?
Q2	Was allocation to treatment groups concealed?
Q3	Were treatment groups similar at baseline?
Q4	Were participants blind to treatment assignment?
Q5	Were those delivering treatment blind to treatment assignment?
Q6	Were outcome assessors blind to treatment assignment?
Q7	Were treatment groups treated identically other than the intervention of interest?
Q8	Was follow-up complete, and if not, were strategies to address incomplete follow-up utilized?
Q9	Were participants analyzed in the groups to which they were randomized?
Q10	Were outcomes measured in the same way for treatment groups?

Q11	Were outcomes measured in a reliable way?
Q12	Was appropriate statistical analysis used?
Q13	Was the trial design appropriate, and any deviations from the standard RCT design (individual randomization, parallel groups) accounted for in the conduct and analysis of the trial?

Items for Case Reports

No.	Question
Q1	Were patient's demographic characteristics clearly described?
Q2	Was the patient's history clearly described and presented as a timeline?
Q3	Was the current clinical condition of the patient on presentation clearly described?
Q4	Were diagnostic tests or assessment methods and the results clearly described?
Q5	Was the intervention(s) or treatment procedure(s) clearly described?
Q6	Was the post-intervention clinical condition clearly described?
Q7	Were adverse events (harms) or unanticipated events identified and described?
Q8	Does the case report provide takeaway lessons?

Items for Cross-Sectional Studies

No.	Question
Q1	Were the criteria for inclusion in the sample clearly defined?
Q2	Were the study subjects and the setting described in detail?
Q3	Was the exposure measured in a valid and reliable way?
Q4	Were objective, standard criteria used for measurement of the condition?
Q5	Were confounding factors identified?
Q6	Were strategies to deal with confounding factors stated?
Q7	Were the outcomes measured in a valid and reliable way?
Q8	Was appropriate statistical analysis used?
